# Clinical Implications of Heart Rate Control in Heart Failure With Atrial Fibrillation: Multi-Center Prospective Observation Registry (CODE-AF Registry)

**DOI:** 10.3389/fcvm.2022.787869

**Published:** 2022-03-22

**Authors:** Shinjeong Song, Jum-Suk Ko, Hye Ah Lee, Eue-Keun Choi, Myung-Jin Cha, Tae-Hoon Kim, Jin-Kyu Park, Jung-Myung Lee, Ki-Woon Kang, Jaemin Shim, Jae-Sun Uhm, Jun Kim, Changsoo Kim, Jin-Bae Kim, Hyung Wook Park, Boyoung Joung, Junbeom Park

**Affiliations:** ^1^Department of Cardiology, College of Medicine, Ewha Womans University School of Medicine, Seoul, South Korea; ^2^Department of Cardiology, Wonkwang University School of Medicine and Hospital, Iksan, South Korea; ^3^Clinical Trial Center, Mokdong Hospital, Ewha Womans University, Seoul, South Korea; ^4^Department of Internal Medicine, Seoul National University Hospital, Seoul, South Korea; ^5^Division of Cardiology, Department of Internal Medicine, Yonsei University College of Medicine, Seoul, South Korea; ^6^Division of Cardiology, Hanyang University Medical College, Seoul, South Korea; ^7^Division of Cardiology, Kyung Hee University Medical College, Seoul, South Korea; ^8^Division of Cardiology, Eulji University Hospital, Daejeon, South Korea; ^9^Division of Cardiology, Korea University Anam Hospital, Seoul, South Korea; ^10^Department of Internal Medicine, University of Ulsan College of Medicine, Seoul, South Korea; ^11^Department of Preventive Medicine, Yonsei University College of Medicine, Seoul, South Korea; ^12^Department of Cardiovascular Medicine, Chonnam National University Medical School, Gwangju, South Korea

**Keywords:** heart failure, atrial fibrillation, rate control, heart failure preserved ejection fraction, U shape curve

## Abstract

**Background:**

Atrial fibrillation (AF) is treated by heart rate (HR) control. However, the optimal HR target in AF patients with heart failure (HF) remains unclear. To evaluate the clinical implication of the resting HR in AF patients with HF accompanied by preserved, mid-range, or reduced ejection fraction (HFpEF, HFmrEF, or HFrEF, respectively).

**Methods:**

Echocardiographic data from June 2016 to April 2020 in a prospective, multicenter, observational registry from 11,104 patients were analyzed. The follow-up duration was 2.2 years. The main outcome was composite of death and hospitalization. We categorized patients according to the HF type and resting HR: ≤ 60 bpm, 61–80 bpm, 81–110 bpm, and >110 bpm.

**Results:**

A total of 1,421 patients were enrolled in the study: 582 in the HFpEF group, 506 in the HFmrEF group, and 333 in the HFrEF group. The patients had a mean age of 69 ± 11 years and consisted of 872 (61.4%) men. Primary endpoint rates among HFpEF patients with 60 < HR ≤ 110 bpm were lower than those with HR ≤ 60 bpm (61–80 bpm group: hazard ratio, 0.66; 95% CI, 0.46–0.94; p = 0.021; 81–110 bpm group: hazard ratio, 0.60; 95% CI, 0.40–0.90; p = 0.013). Especially, HFpEF patients with HR 81–110 bpm had a lower incidence of hospitalization caused by HF aggravation than those with other HR strata (HR ≤ 80bpm strata or HR >110 bpm strata). In HFmrEF and HFrEF patients, the survival rates did not differ significantly among patients in the three groups with HR ≤ 110 bpm. Moreover, the event rates increased significantly in HFmrEF patients with HR >110 bpm (hazard ratio, 1.91; 95% CI, 1.16-3.14, p = 0.011).

**Conclusion:**

In patients with AF and HFpEF, the resting HR has U-shaped associations with the overall primary endpoint. A lower or higher resting HR is associated with increased cardiovascular outcomes, especially in patients with HFpEF and AF.

## Introduction

Heart failure (HF) is commonly accompanied by atrial fibrillation (AF) irrespective of the concomitant left ventricular ejection fraction (LVEF) (1). Rhythm control in patients with HF and AF is associated with lower mortality and morbidity (2); however, the optimal heart rate (HR) target in AF patients with HF remains unclear. According to the 2016 ESC Guidelines for the diagnosis and treatment of acute and chronic HF, the estimated HR is 60–110 bpm (3). In the RACE II trial, lenient rate control (>110 bpm) was as effective as strict rate control in persistent AF patients (4). However, this study did not include patients with HF (the average LVEF was approximately 52%), and the incidence of a previous hospitalization for HF was only about 10%.

In HF patients without AF, the target for an appropriate HR has not been specified. In patients with an HR ≥70 bpm taking the maximally tolerated dose of beta-blockers, ivabradine was recommended (5). In contrast to sinus rhythm, slower HRs are not associated with survival benefits in AF (6). The irregular rhythm of AF also has detrimental effects on systolic and diastolic heart function independent of HR (7, 8). These factors may explain why titration using a beta-blocker fails to reduce mortality or morbidity in AF, unlike in sinus rhythm.

HF with preserved ejection fraction is as common as the syndrome with reduced LVEF (9); however, the target HR in patients with HF with preserved ejection fraction (HFpEF) and AF is not clear. In this prospective, multicenter, observational study (in 12 tertiary hospitals in Korea), we investigated the clinical characteristics and implications of the resting HR in AF patients with HF, for the three subtypes of HF defined by the LVEF.

## Methods

### Study Protocol

In a prospective observational registry (COmparison study of Drugs for symptom control and complication prEvention of Atrial Fibrillation [CODE-AF] registry), 11,104 patients with non-valvular AF were consecutively enrolled between June 2016 and April 2020 from 12 tertiary centers in Korea (10). All patients were >18 years old. After enrollment, each patient was followed up every 6 months, either through the outpatient clinic or by telephone contact. This study was conducted in accordance with the Declaration of Helsinki and the relevant guidelines and regulations. The study protocol was approved by the Research Ethics Committee of all 12 tertiary centers including Ewha Womans University Mokdong Hospital (No. 216-02-056), and all patients provided their written informed consent prior to enrollment. This study was approved by the ethics committee of each center, and the study is registered at www.ClinicalTrials.gov (NCT 02786095).

**Figure 1:**
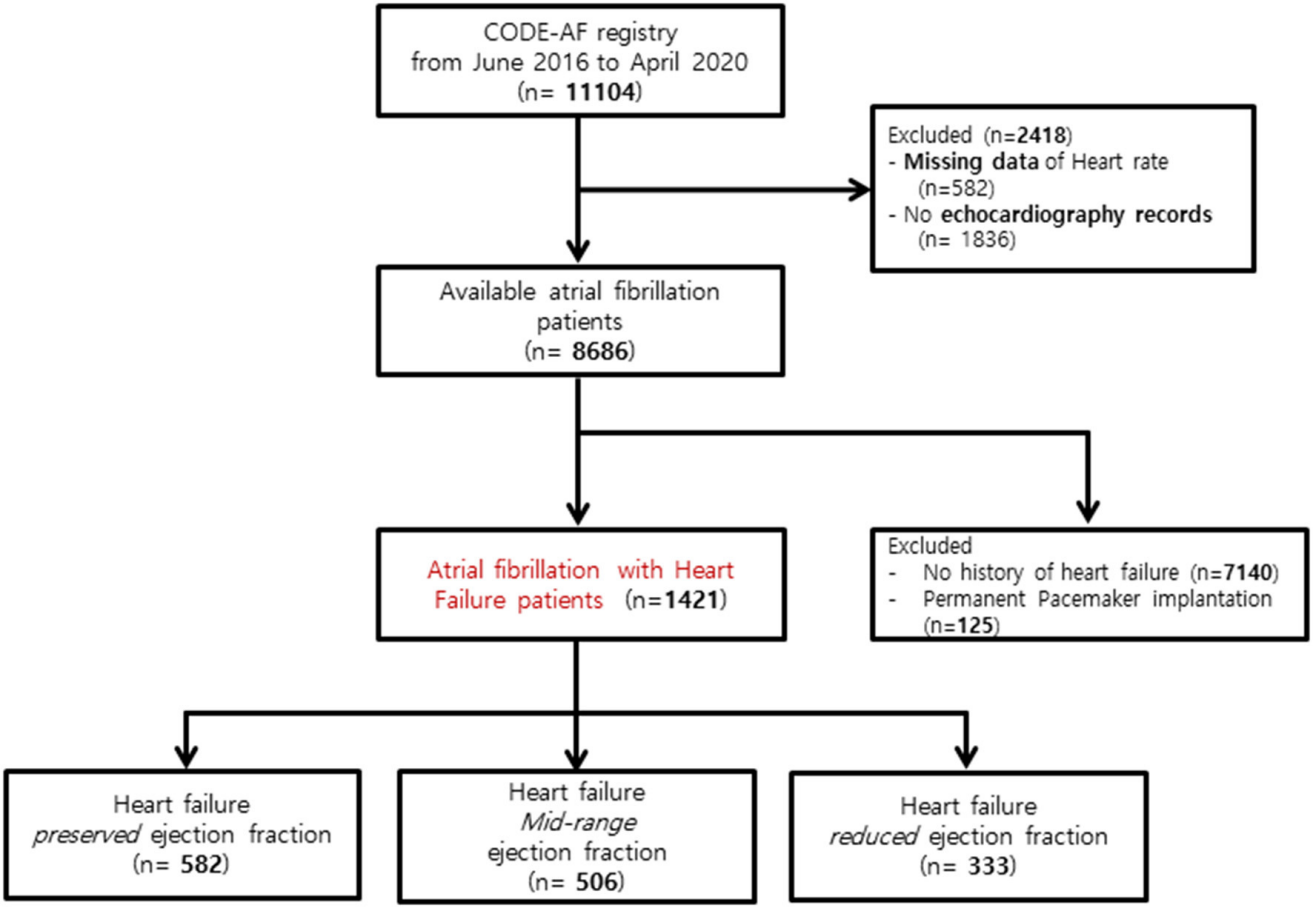
Flowchart of the study population enrollment. CODE-AF, COmparison study of Drugs for symptom control and complication prEvention of Atrial Fibrillation.

Among the 11,104 participants enrolled in CODE-AF, we excluded 2,418 patients for missing data on HR (*n* = 582) and missing echocardiography records (*n* = 1,836). Among available AF patients with available data, patients without a history of HF (*n* = 7,140) or with a permanent pacemaker (*n* = 125) were also excluded (Figure 1).

### Definitions Used

The recent guidelines classify patients with HF in three categories: HF with preserved ejection fraction (HFpEF), defined by an LVEF ≥50%, HF with reduced ejection fraction (HFrEF) if the LVEF is <40%, and HF with mid-range ejection fraction (HFmrEF) if the LVEF is 40–49% (11). Considering the echocardiogram results and history of heart failure at the time of AF enrollment, the patients were diagnosed with HF. As suggested in the recent guidelines, we classified 1,421 patients with HF history in this study into HFpEF, HFmrEF, and HFrEF.

### Baseline Covariates

We collected data on the clinical characteristics, medical history, comorbidities, blood pressure, medications, therapies, and interventions. Systolic and diastolic blood pressure and CHA2DS2-VASc score were included as continuous variables in the Cox regression models. Echocardiographic variables such as left atrial size, left atrial volume index, LVEF, and E/e' were collected.

In addition to other baseline variables, we recorded the stable HR before the deterioration and HR measured by electrocardiogram at discharge from the hospital or outpatient visits at the cardiology department. In paroxysmal AF patients, the heart rate was checked using EKG documented an atrial fibrillation rhythm. The HR was categorized into four strata and analyzed by a Cox regression model, the event rates, and hazard ratio: ≤ 60, 61–80, 81–110, and >110 bpm.

### Outcomes

The primary outcome of this study was a composite of all-cause mortality and hospitalizations. A subgroup analysis of the primary outcome was also performed. The secondary outcomes of this study were composite clinical events including stroke, systemic embolism, major bleeding, myocardial infarction, and arrhythmic events (syncope, sustained ventricular tachycardia, and cardiac arrest). A stroke was defined as the sudden onset of a focal deficit consistent with an occlusion of a major cerebral artery (documented by imaging). Systemic embolisms were defined as an acute vascular occlusion of an extremity or organ as documented by imaging. Major bleeding was defined as a reduction in the hemoglobin level by at least 20 g/L, a transfusion of at least 2 units of blood, or symptomatic bleeding in a critical area or organ. Syncope was defined as a transient loss of consciousness that may have been caused by an arrhythmia. Sustained ventricular tachycardia was defined as hemodynamic compromised ventricular tachycardia lasting more than 30 s or requiring electrical termination. Cardiac arrest was defined as circulatory arrest requiring resuscitation and hospitalization (6). All-cause mortality and major adverse outcomes described above were checked by patient interviews and medical records during follow-up.

### Statistical Analysis

Continuous variables are presented as the mean ± SD, whereas categorical variables are presented as counts and percentages. Comparisons of the variables across the groups were performed using Student's *t* test or a one-way ANOVA combined with a Bonferroni *post hoc* analysis for continuous variables and Chi-square (χ2) or Fisher's exact test for categorical variables, as appropriate. The primary analysis for the primary endpoint consisted of a comparison among the HFpEF, HFmrEF, and HFrEF groups for the time to the first occurrence of the composite primary outcome as assessed through Kaplan–Meier curves and by performing log-rank tests among the HR strata. Cox proportional hazards models were used to calculate the test hazard ratios in the HFpEF, HFmrEF, and HFrEF subgroups. The covariates used in all adjusted Cox models were clinical characteristics, medical history, comorbidity, blood pressure, medication, therapy, and intervention. We first constructed a baseline Cox model that included variables mentioned above. A penalized spline term for parity was used upon further examination of the data. We evaluated the model for proportional hazards assumptions, influential observations, and nonlinearity of continuous variables. The final model was fitted using the smoothHR package of R software (12) that estimates log hazard ratios and corresponding confidence intervals for nonlinear continuous variables. Statistical analyses were performed using the SPSS version 22.0 software package (IBM SPSS, NY, USA) and R version 3.6.2 smoothHR software package. A *p* < 0.05 was considered to indicate statistical significance.

## Results

### Baseline Characteristics

**Figure 2:**
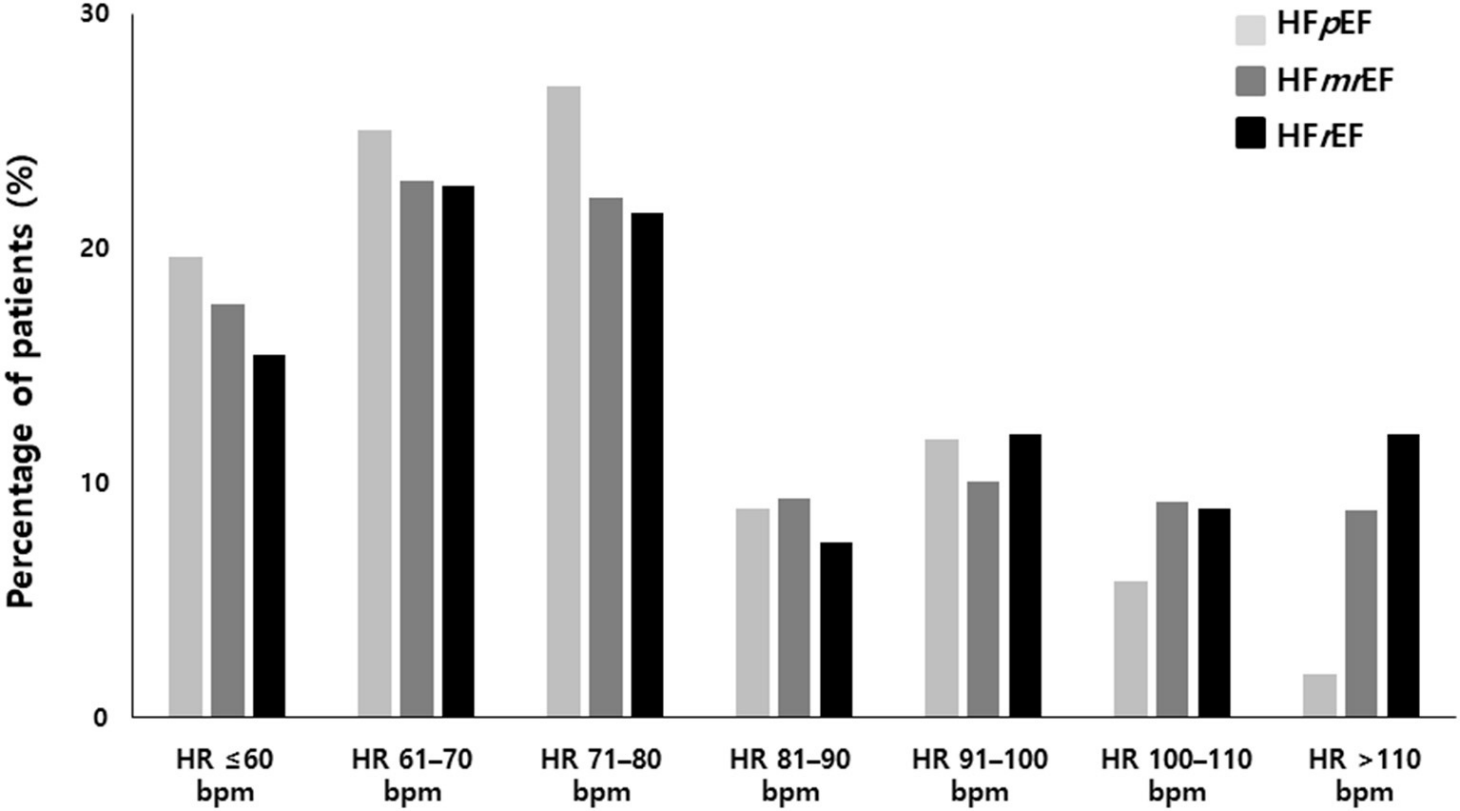
Heart rate distribution in HFpEF, HFmrEF and HFrEF. HFpEF, heart failure with preserved ejection fraction; HFmrEF, heart failure with mid-range ejection fraction; HFrEF, heart failure with reduced ejection fraction.

A total of 1,421 patients were enrolled in the study: 582 in the HFpEF group, 506 in the HFmrEF group, and 333 in the HFrEF group (Table 1 and Figure 1). The baseline characteristics of the study population are listed in Table 1. The patients had a mean age of 69 ± 11 years and consisted of 872 (61.4%) men. The mean CHA2DS2-VASc score was 3.4 ± 1.7, and 34% patients had persistent AF. The distribution of patients with HFpEF, HFmrEF, and HFrEF according to the resting HR is shown in Figure 2. The percentage of women (54%), valvular disease (15%), PeAF (38%) and prevalence of hypertension (75%) in the HFpEF group were higher than those in the HFmrEF and HFrEF groups. Valvular disease distribution based on ejection fraction is shown in Supplementary Material. However, there were more patients with a history of myocardial infarction (10%) and implantable cardioverter defibrillator insertion (12%) in the HFrEF group than in the HFpEF and HFmrEF groups.

**Table 1:** Baseline characteristics based on the ejection fraction.

	**HFpEF** **(*N* = 582)**	**HFmrEF (*N* = 506)**	**HFrEF** **(*N* = 333)**	***P*** **value**
Age, years	71 ± 10	68 ± 11	67 ± 11	<0.001
Male	270 (46)	361 (71)	241 (72)	<0.001
Persistent AF, n (%)	217 (38)	173 (34)	97 (29)	0.019
CHA_2_DS_2_ VASc	3.7 ± 1.6	3.1 ± 1.8	3.4 ± 1.8	<0.001
Follow up duration, days				
Median	894	778	689	0.005
Interquartile range	351-1183	356-1112	267-1099	
SBP, mmHg	123 ± 16	118 ± 17	118 ± 17	<0.001
DBP, mmHg	74 ± 13	74 ± 13	74 ± 14	0.788
**Medical history**, n (%)				
Hypertension	437 (75)	325 (64)	211 (63)	<0.001
Diabetes mellitus	153 (26)	138 (27)	113 (34)	0.038
Myocardial infarct	11 (2)	45 (9)	33 (10)	<0.001
Valvular disease	86 (15)	46 (9)	25 (8)	0.001
Valvular surgery	1 (0)	0 (0)	1 (0)	0.508
ICD	13 (2)	9 (2)	40 (12)	<0.001
Chronic kidney disease	85 (15)	75 (15)	49 (15)	0.995
Smoker	134 (23)	148 (29)	124 (37)	<0.001
**Rate-control medications in use no. (%)**				
None	209 (36)	144 (28)	97 (29)	0.016
Beta-blocker alone	251 (43)	3188 (37)	122 (37)	0.064
Verapamil or diltiazem alone	65 (11)	82 (16)	51 (15)	0.040
Digoxin alone	8 (1)	16 (3)	6 (2)	0.112
Beta-blocker and CCB	35 (6)	51 (10)	42 (13)	0.002
Beta-blocker and digoxin	7 (1)	15 (3)	11 (3)	0.062
Digoxin and CCB	7 (1)	5 (1)	1 (0)	0.378
Beta-blocker, digoxin, CCB	0	5 (1)	3 (1)	0.061
**Other medications in use at baseline**				
Warfarin	118 (20)	74 (15)	63 (19)	0.046
NOAC	362 (62)	335 (66)	222 (67)	0.265
ARB or ACEi	310 (53)	264 (52)	232 (70)	<0.001
Statin	224 (38)	194 (38)	122 (37)	0.841
Antiplatelet	95 (16)	83 (16)	64 (19)	0.421
Diuretics	70 (12)	35 (7)	23 (7)	0.002
**Echocardiographic variables**				
Left atrial size, long axis, mm	49 ± 8	46 ± 8	48 ± 9	0.029
Left atrial volume index, ml/m^2^	58 ± 26	56 ± 28	62 ± 28	0.035
LV ejection fraction, %	61 ± 7	45 ± 3	31 ± 6	<0.001
E/e'	15 ± 7	13 ± 6	16 ± 9	<0.001

*HFpEF, Heart failure preserved ejection fraction; HFrEF, Heart failure reduced ejection fraction; bpm, beat per minute; AF, atrial fibrillation; SBP, systolic blood pressure; DBP, diastolic blood pressure; ICD, implantable cardioverter-defibrillator; LV, left ventricle; CCB, calcium channel blocker (verapamil or diltiazem); NOAC, novel oral anticoagulants; ARB, angiotensin-receptor blockers; ACEi, angiotensin-converting-enzyme inhibitors*.

### Outcomes Related to the EF

The median follow-up was 2.2 (0.9–3.1) years. The primary endpoint occurred in 219 (17.4% per person-year) patients with HFpEF, 197 (19.2% per person-year) patients with HFmrEF, and 145 (22.9% per person-year) patients with HFrEF. The primary endpoint did not differ significantly among the groups. However, the secondary endpoint events were significantly more frequent in the HFmrEF (45; 4.4% per person-year) and HFrEF (28; 4.4% per person-year) groups than in the HFpEF group (17; 1.3% per person-year, *p* < 0.001) (**Tables 2, 3**).

### Primary Outcomes Related to the HR

**Figure 3:**
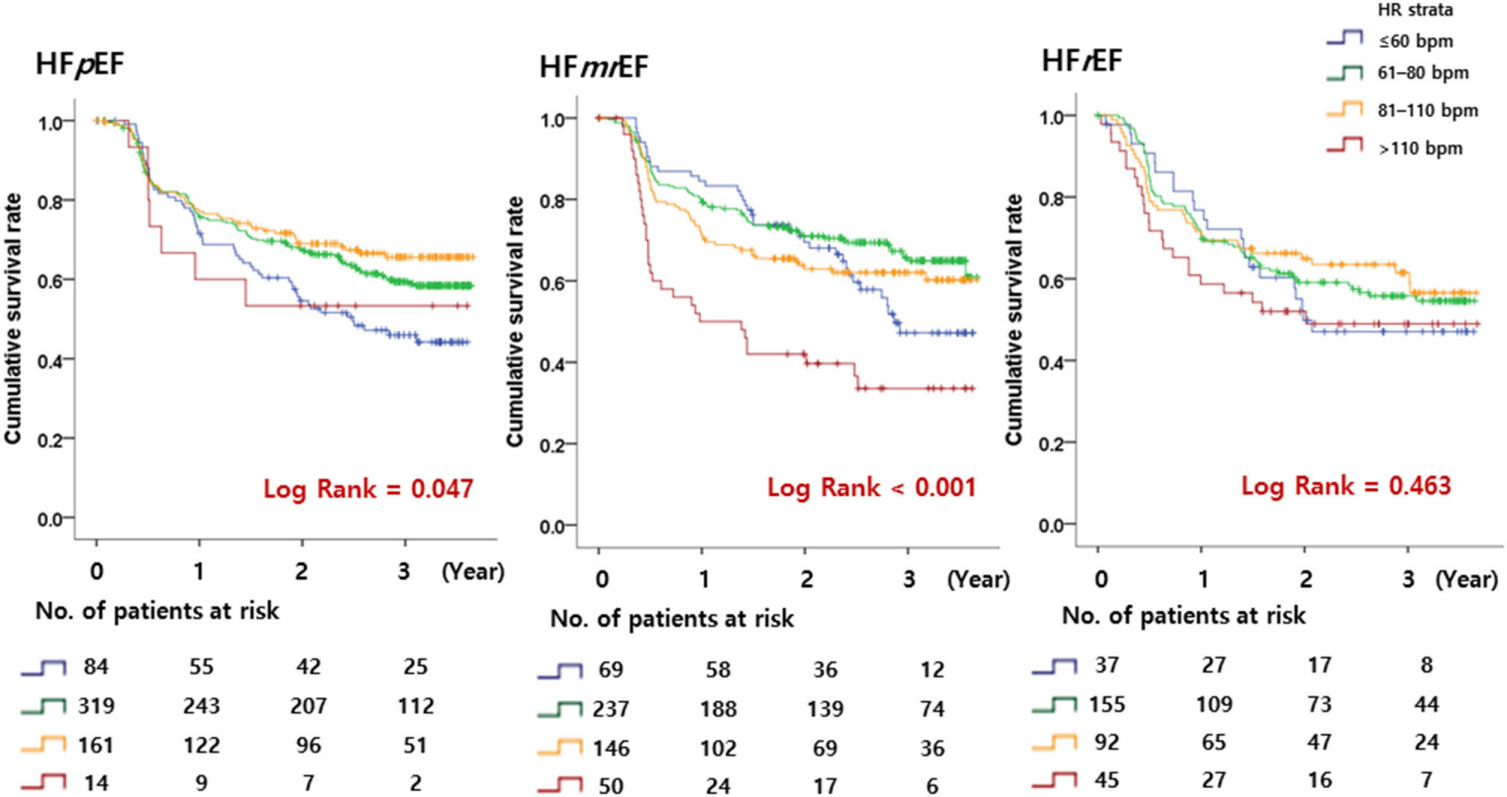
Kaplan–Meier curves. The primary endpoint according to the heart rate in patients with HFpEF, HFmrEF, and HFrEF is shown. HFpEF, heart failure with preserved ejection fraction; HFmrEF, heart failure with mid-range ejection fraction; HFrEF, heart failure with reduced ejection fraction.

A total of 561 patients (19.2% per person-year) reached the primary outcome. Kaplan–Meier curves for the primary outcome are shown in Figure 3. In a multivariable cox regression analysis using baseline data corrected for age, sex, weight, AF type, CHA2DS2 VASc score, HR, systolic blood pressure, diastolic blood pressure, medical history, and medication in use, the survival rates among HFpEF patients with 60 < HR ≤ 110 bpm were lower than those with HR ≤ 60 bpm (61–80 bpm group: hazard ratio, 0.66; 95% CI, 0.46–0.94; *p* = 0.021; 81–110 bpm group: hazard ratio, 0.60; 95% CI, 0.40–0.90; *p* = 0.013) (Table 2). In HFmrEF and HFrEF patients, the survival rates did not differ significantly among patients in the three groups with HR ≤ 110 bpm. Moreover, the event rates increased significantly in HFmrEF patients with HR >110 bpm (hazard ratio, 1.91; 95% CI, 1.16–3.14, *p* = 0.011). Especially, HFpEF patients with HR 61–110 bpm had a lower incidence of hospitalization caused by HF aggravation than those with other HR strata (HR ≤ 60 bpm strata or HR >110 bpm strata) (In HR strata 61–80; hazard ratio, 0.70; 95% CI, 0.51–0.97; p = 0.030, In HR strata 81–110 hazard ratio, 0.58; 95% CI, 0.39–0.85; p=0.005) (Table 2). Moreover, HFmrEF patients with HR >110 bpm had significantly higher rate of hospitalization caused by HF (hazard ratio, 1.99; 95% CI, 1.18–3.36; *p* = 0.010).

**Table 2:** Cox regression analyses of the effect of the heart rate on the primary endpoint.

**HR strata, beats per min**		**No. of events**	**Hazard ratio (95% CI)**	***P*** **value**
**Composite primary endpoint**				
In HFpEF	≤ 60 bpm	42	Reference	
(*n =* 219, 17.4%/person-year)	61–80 bpm	118	**0.66 (0.46–0.94)**	**0.021**
	81–110 bpm	52	**0.60 (0.40–0.90)**	**0.013**
	>110 bpm	7	0.97 (0.44–2.11)	0.944
In HFmrEF	≤ 60 bpm	31	Reference	
(*n =* 197, 19.2%/person-year)	61–80 bpm	79	0.71 (0.47–1.08)	0.106
	81–110 bpm	56	0.89 (0.57–1.37)	0.587
	>110 bpm	31	**1.91 (1.16–3.14)**	**0.011**
In HFrEF	≤ 60 bpm	19	Reference	
(*n =* 145, 22.9%/person-year)	61–80 bpm	67	0.70 (0.42–1.19)	0.188
	81–110 bpm	36	0.61 (0.34–1.10)	0.099
	>110 bpm	23	0.93 (0.49–1.77)	0.827
**Individual components**				
**death**				
HFpEF	≤ 60 bpm	2	Reference	
(*n =* 15, 1.2%/person-year)	61–80 bpm	7	–	
	81–110 bpm	5	–	-
	>110 bpm	1	–	-
In HFmrEF:	≤ 60 bpm	1	Reference	
(*n =* 7, 0.7%/person-year)	61–80 bpm	3	–	
	81–110 bpm	3	–	
	>110 bpm	0	–	
HFrEF:	≤ 60 bpm	0	Reference	
(*n =* 9, 1.4%/person-year)	61–80 bpm	4	–	
	81–110 bpm	2	–	
	>110 bpm	3	–	
**Hospitalization due to an HF aggravation**				
HFpEF	≤ 60 bpm	41	Reference	
(*n =* 216, 17.2%person-year)	61–80 bpm	118	0.70 (0.51–0.97)	0.030
	81–110 bpm	51	0.58 (0.39–0.85)	0.005
	>110 bpm	6	0.87 (0.37–2.01)	0.738
In HFmrEF:	≤ 60 bpm	26	Reference	
(*n =* 180, 17.5%erson-year)	61–80 bpm	73	0.83 (0.55–1.27)	0.398
	81–110 bpm	55	1.09 (0.70–1.70)	0.696
	>110 bpm	26	1.99 (1.18–3.36)	0.010
HFrEF:	≤ 60 bpm	17	Reference	
(*n =* 128, 20.2%/person-year)	61–80 bpm	57	0.66 (0.40–1.07)	0.092
	81–110 bpm	33	0.63 (0.36–1.09)	0.100
	>110 bpm	21	1.06 (0.58–1.94)	0.855
**Hospitalization due to other causes**				
HFpEF	≤ 60 bpm	2	Reference	
(*n =* 5, 0.4%person-year)	61–80 bpm	0	0.00 (0.01–4.01)	0.944
	81–110 bpm	2	0.61 (0.07–5.35)	0.652
	>110 bpm	1	8.21 (0.47–94.0)	0.149
In HFmrEF:	≤ 60 bpm	5	Reference	
(*n =* 17, 1.7%/person-year)	61–80 bpm	6	0.32 (0.10–0.99)	0.048
	81–110 bpm	1	0.10 (0.01–0.82)	0.032
	>110 bpm	5	2.00 (0.61–6.57)	0.253
HFrEF	≤ 60 bpm	2	Reference	
(*n =* 17, 2.7%/person-year)	61–80 bpm	10	0.91 (0.19–4.42)	0.904
	81–110 bpm	3	0.41 (0.06–2.76)	0.362
	>110 bpm	2	0.61 (0.08–4.92)	0.640

*A multivariable Cox regression was performed with the variables listed in Table 1*.

*CI, confidence interval; HF, heart failure; HR, heart rate; HFpEF, heart failure preserved ejection fraction; HR, heart rate; HFrEF, heart failure reduced ejection fraction; bpm, beat per minute*.

*Bold values mean statistically significant value (p < 0.05)*.

**Figure 4:**
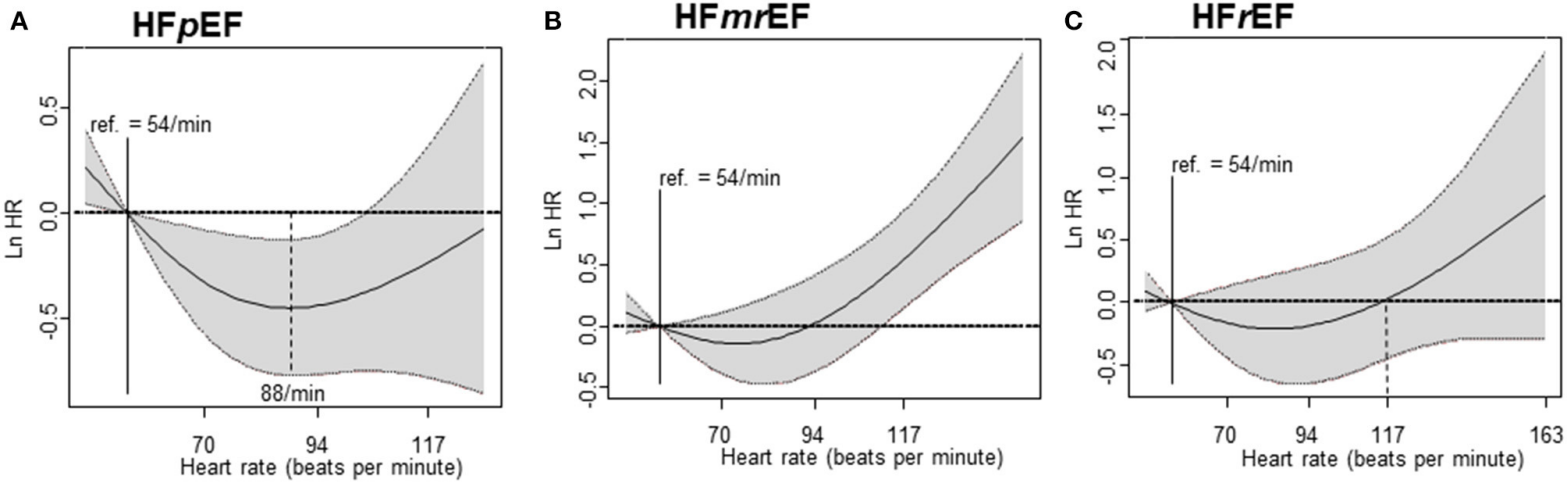
Cox regression analysis – penalized spline. The primary endpoint according to heart failure in patients with HFpEF, HFmrEF, and HFrEF is shown. **(A)** Penalized spline regression in HFpEF, **(B)** Penalized spline regression in HFmrEF, and **(C)** Penalized spline regression in HFrEF. HFpEF, heart failure with preserved ejection fraction; HFmrEF, heart failure with mid-range ejection fraction; HFrEF, heart failure with reduced ejection fraction.

**Figure 5:**
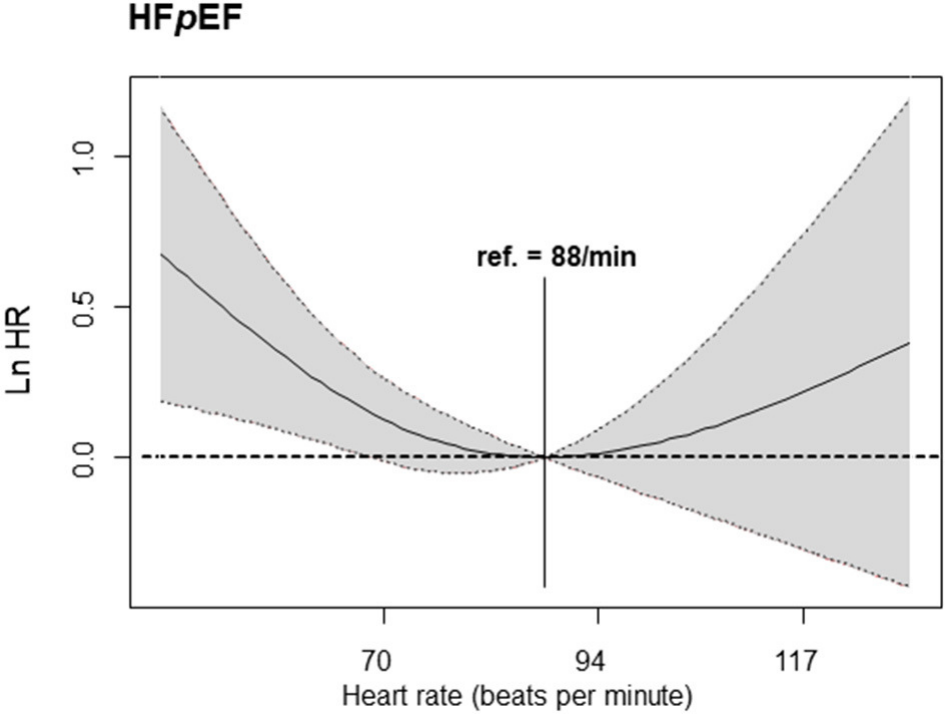
Cox regression analysis—penalized spline. The primary endpoint according to heart failure in patients with HFpEF (reference heart rate of 88 bpm) is shown. HFpEF, heart failure with preserved ejection fraction.

The penalized spline for the composite primary outcome showed the minimum hazard in the HR 80–110 bpm strata (HR = 88) among HFpEF patients. Similar to the results of our cox regression analysis, the hazard curve in HFmrEF patients gradually increased to the right in the HR >110 bpm strata. In HFrEF patients, the hazard curve did not show any significant increase or decrease depending on the HR (Figure 4). The penalized spline for parity resulted in a U-shaped hazard curve with the minimum hazard (i.e., composite primary endpoint—all-cause mortality and all-cause hospitalization) in the group with HR = 88 bpm (Figure 5).

Considering gender, age, diastolic blood pressure, and ICD as correction variables, the difference in the influence on primary outcomes between HF groups (HFpEF vs. HFmrEF vs. HFrEF) according to the HR strata was not statistically significant (*p* = 0.7827). Also, the difference in the influence on primary outcomes between AF types according to the HR strata was not statistically significant (*p* =0.395).

### Secondary Outcomes Related to the HR

In the HFpEF group, the secondary outcome rate was significantly lower than that in the other two groups. However, the secondary outcome did not differ significantly according to HR strata within each HF group. The cumulative incidences of the components of the secondary outcome are shown in Table 3.

**Table 3:** Cox regression analyses of the effect of the heart rate on the secondary endpoint.

**HR strata, beats per min**		**No. of events**	**Hazard ratio**	***P*** **value**
		**(% per person-year)**	**(95% CI)**	
**Composite secondary endpoint**				
In HFpEF	≤ 60 bpm	2	Reference	
(*n* = 17, 1.3%/person-year)	61–80 bpm	8	0.64 (0.13–3.25)	0.591
	81–110 bpm	6	1.44 (0.28–7.58)	0.664
	>110 bpm	1	4.88 (0.37–64.75)	0.230
In HmFrEF:	≤ 60 bpm	8	Reference	
(*n* = 45, 4.4%/person-year)	61–80 bpm	21	0.50 (0.21–1.22)	0.127
	81–110 bpm	8	0.44 (0.16–1.19)	0.105
	>110 bpm	8	2.78 (0.87–8.90)	0.086
In HFrEF:	≤ 60 bpm	2	Reference	
(*n* = 28, 4.4%/person-year)	61–80 bpm	18	1.75 (0.41–7.59)	0.453
	81–110 bpm	6	0.91 (0.18–4.64)	0.909
	>110 bpm	2	0.55 (0.07–4.22)	0.561

*A multivariable Cox regression was performed with the variables listed in Table 1 without echoparameters*.

*CI, confidence interval; HF, heart failure; HR, heart rate; HFpEF, heart failure preserved ejection fraction; HR, heart rate; HFrEF, heart failure reduced ejection fraction; bpm, beat per minute*.

## Discussion

In this study, we observed a U-shaped association between the resting HR and cardiovascular outcomes in patients with HFpEF and AF, with the lowest event rates observed in patients with HR 81–110 bpm. However, the cardiovascular outcome rate was similar among HFmrEF patients with HR ≤ 110 bpm, and the rate increased only in patients with a higher resting HR (HR >110 bpm). The optimal HR in HF in patients with AF has been studied previously. *Post-hoc* analysis of the RACE II study showed that a lenient HR was non-inferior in patients with HF and AF compared to a strict HR control. The average EF in that study was approximately 47–48% (13), which is comparable to the 45% observed in the HFmrEF group in our study. In the HFmrEF group in our study, there was no difference in the cardiovascular outcomes in patients with HR ≤ 110 bpm, which is similar to the result of the previous study. Similarly in the HFrEF group, there was no statistical significance, but the tendency of HR to increase as the resting heart rate increased was shown. The tendency of HFrEF group shows similar conclusion to previous study; rate control using b-blockers should not be used preferentially to improve prognosis in patients with concomitant heart failure and atrial fibrillation (14). Actually, EF in the previous study group is similar to EF in this HFrEF group (average EF; 23 vs. 31%). The previous study did not analyze HFpEF patients alone, whereas in our study, we classified and analyzed HFpEF patients separately. We found a U shape association of the resting HR to the cardiovascular outcomes in HFpEF patients with AF, indicating increased adverse cardiovascular outcomes with low or high HRs.

AF is commonly observed in HF irrespective of the concomitant LVEF (1). The prevalence of HF is increasing, and older age, non-cardiac comorbidities, higher rates of AF, and limited treatment options further complicate the management of HF cases. Since these patients are often elderly and highly symptomatic and often have a poor quality of life (15), it is important to alleviate symptoms, improve wellbeing, and reduce hospitalization (16). There is no consensus on treatments that can effectively improve the clinical outcomes in HFpEF patients. However, HFrEF is more commonly associated with coronary artery disease and can be treated with evidence-based therapies. A previous study suggested that a lower resting HR is associated with better survival in HFrEF patients with sinus rhythm but not in those with AF (6). HFpEF and AF are common coexistent conditions that have a substantial impact on the patients' wellbeing. The prevalence of HFpEF in four major AF trials (17) has been 8–24%. The difference in the HF incidence in each trial may be due to the different AF types included in each trial. In addition, the prevalence of AF in HFpEF varied among seven large HF trials, ranging from 21 to 33% (17). AF is a strong and independent prognostic factor in HF patients and has been shown in clinical trials and observational studies to increase the risk of death (14, 18). Both HFpEF and AF share similar clinical aspects and are associated with aging, hypertension, and diastolic dysfunction. Thus, they affect each other's adverse cardiovascular outcomes (19). Some guidelines have suggested that the optimal resting HR in HF patients may be 60–110 bpm (20–23). Strict HR control is associated with a worse outcome (24). Patients with HF and AF exhibit structural changes that cause AF (e.g., an enlarged left atrium (LA), LA to LV flow during early diastolic phase, and loss of A waves), making it more difficult to determine the appropriate HR because of the relationship between these two vicious twins (17, 25, 26).

A previous study in AF patients showed that a lenient rate control is as effective as a strict rate control in terms of major clinical events (6). The patients included in the study showed a preserved EF of about 52% in both the lenient control and strict control groups, with about 15% of the patients having an EF under 40%. In a *post-hoc* analysis, in about 32% patients with reduced EF, the overall average EF was 47–48%, mostly referred to as HFpEF. However, this association of HF with AF was observed when there was no strict classification for HFpEF and HFrEF.

A low HR is beneficial for both patients with HF (27, 28) and those with coronary artery disease (29). However, this presumption is primarily derived from studies in patients with sinus rhythm. A major difference in the evaluation of the diastolic function between patients with AF and those with sinus rhythm is the elimination of A waves seen in the late diastolic phase, i.e., “loss of A waves”. In patients with sinus rhythm with a relaxation abnormality, an increase in diastolic phase due to reduced HR will assist in LV filling, thereby maintaining the cardiac output, increasing the coronary blood supply, and alleviating the symptoms. However, in cases of severe diastolic dysfunction, an increase in the diastolic phase due to reduced HR will not significantly improve the cardiac output because LV filling is dependent on only the HR (especially for restrictive patterns). In AF patients, LV filling is largely dependent on the E wave acceleration time and LV diastolic function, since there are no A waves. Therefore, if the rate is adjusted strictly in patients with diastolic dysfunction AF, the filling from LA to LV is reduced, and the flexibility of the LA is reduced due to AF, thus increasing the pulmonary congestion. This worsens the symptoms and deteriorates the general condition, which marks the beginning of a vicious cycle that causes uncontrolled AF. This hypothesis is consistent with the notion that beta-blockers do not improve the prognosis in patients with HF and AF (30, 31).

Our study has several limitations. First, the diagnosis of HF may have been affected by the variability among clinicians since HF is a clinical syndrome and the diagnosis is mainly based on the presence of typical symptoms and signs rather than specific test results (2). Sometimes, it can be difficult to distinguish which symptoms are caused by heart failure or arrhythmia, as determined by clinicians. This could explain why the heart failure rate is lower in our cohort compared to other studies (13, 32–34). Second, although the HR is clinically determined to be in the resting state during hospitalization in the outpatient clinic, this is only a single measure, and we could not obtain information on the HR variability. Third, as this study was conducted with a relatively short follow-up period (median 2.2 years), the number of adverse events cases is small. Also, follow-up echocardiographic data are not available. So the proportion of patients with reversible LV dysfunction in the HFrEF group could not be determined. This could have resulted in the lower than predicted adverse outcome rates about death especially in the HFrEF group. Finally, this study was conducted in a single country; therefore, the application of our results to other regions requires more study.

## Conclusion

In patients with HF and AF, a high HR (>110 bpm) is associated with an increased risk of cardiac adverse events compared with an HR ≤ 110 bpm regardless of the type of HF. Importantly, a very low HR (≤ 60 bpm) in patients with HFpEF and AF is also associated with an increased risk of U-shaped cardiac adverse events.

## Data Availability Statement

The datasets presented in this study can be found in online repositories. The names of the repository/repositories and accession number(s) can be found below: COmparison study of Drugs for symptom control and complication prEvention of Atrial Fibrillation [CODE-AF] registry.

## Ethics Statement

The studies involving human participants were reviewed and approved by Research Ethics Committee of all 12 tertiary centers including Ewha Womans University Mokdong Hospital (No. 216-02-056). The patients/participants provided their written informed consent to participate in this study.

## Author Contributions

SS and JP contributed to conception and design of the study. J-SK, E-KC, M-JC, T-HK, J-KP, J-ML, K-WK, J-SU, JK, CK, J-BK, HP, and BJ organized the database. HL performed the statistical analysis. SS wrote the first draft of the manuscript. All authors contributed to manuscript revision, read, and approved the submitted version.

## Funding

This study was supported by a research grant from the Korean Healthcare Technology R&D project funded by the Ministry of Health and Welfare (HI15C1200, HC19C0130). This research was also supported by the Basic Science Research Program through the National Research Foundation of Korea (NRF) funded by the Ministry of Science, ICT and Future Planning (NRF-2017R1E1A1A01078382).

## Conflict of Interest

BJ has served as a speaker for Bayer, BMS/Pfizer, Medtronic, and Daiichi-Sankyo and has received research funds from Medtronic and Abbott. The remaining authors declare that the research was conducted in the absence of any commercial or financial relationships that could be construed as a potential conflict of interest.

## Publisher's Note

All claims expressed in this article are solely those of the authors and do not necessarily represent those of their affiliated organizations, or those of the publisher, the editors and the reviewers. Any product that may be evaluated in this article, or claim that may be made by its manufacturer, is not guaranteed or endorsed by the publisher.
