# Supplementary material for: Clinical Implications of Heart Rate Control in Heart Failure With Atrial Fibrillation: Multi-Center Prospective Observation Registry (CODE-AF Registry)
Source: Front Cardiovasc Med. 2022 Mar 22;9:787869. doi: 10.3389/fcvm.2022.787869 (PMC8980522; doi:10.3389/fcvm.2022.787869)
Supplement: Supplementary file 1 [file Table_1.docx]

|  | HFpEF (73) | HFmrEF (47) | HFrEF (37) |
| --- | --- | --- | --- |
| Aortic regurgitation | 10 | 12 | 7 |
| Aortic stenosis | - | 2 | 1 |
| Mitral regurgitation | 40 | 24 | 12 |
| Mild mitral stenosis | 1 |  | 1 |
| Tricuspid regurgitation | 49 | 30 | 25 |

**Supplementary Table. Valvular disease distribution based on ejection fraction**

*Even if one patient had various valve diseases, it was counted by dividing it into each valve disease.
